# Co-Carriage of *bla*_KPC-2_ and *bla*_NDM-1_ in Clinical Isolates of *Pseudomonas aeruginosa* Associated with Hospital Infections from India

**DOI:** 10.1371/journal.pone.0145823

**Published:** 2015-12-29

**Authors:** Deepjyoti Paul, Debadatta Dhar Chanda, Anand Prakash Maurya, Shweta Mishra, Atanu Chakravarty, Gauri Dutt Sharma, Amitabha Bhattacharjee

**Affiliations:** 1 Department of Microbiology, Assam University, Silchar, India; 2 Department of Microbiology, Silchar Medical College and Hospital, Silchar, India; 3 Department of Microbiology, Institute of Medical Sciences, Banaras Hindu University, Varanasi, India; 4 Department of Life science and Bioinformatics, Assam University, Silchar, India; Laurentian, CANADA

## Abstract

Global spread of KPC poses to be a serious threat complicating treatment options in hospital settings. The present study investigates the genetic environment of *bla*_KPC-2_ among clinical isolates of *Pseudomonas aeruginosa* from a tertiary referral hospital of India. The study isolates were collected from different wards and clinics of Silchar Medical College and Hospital, India, from 2012–2013. The presence of *bla*_KPC_ was confirmed by genotypic characterization followed by sequencing. Cloning of the *bla*_KPC-2_ gene was performed and the genetic environment of this gene was characterized as well. Transferability of the resistance gene was determined by transformation assay and Southern hybridization. Additionally, restriction mapping was also carried out. Two isolates of *P*. *aeruginosa* were found to harbor *bla*_KPC-2_, were resistant towards aminoglycosides, quinolone and β-lactam-β-lactamase inhibitor combination. In both the isolates, the resistance determinant was associated with class 1 integron and horizontally transferable. Both the isolates were co-harboring *bla*_NDM-1_. The first detection of this integron mediated *bla*_KPC-2_ coexisting with *bla*_NDM-1_ in *P*. *aeruginosa* from India is worrisome, and further investigation is required to track the gene cassette mediated *bla*_KPC-2_ in terms of infection control and to prevent the spread of this gene in hospitals as well as in the community.

## Introduction

*Klebsiella pneumoniae* carbapenemase (KPC) is a major contributor for carbapenem resistance across the globe within the members of Enterobacteriaceae [1] and in recent past they are also been reported in *Pseudomonas* spp. from Brazil and China [2, 3]. In Indian subcontinent, this gene has so far been reported only in Enterobacteriaceae family [4]. Their coexistence has been witnessed with other carbapenemase genes as well [4]. However, no study from this country has attempted to investigate genetic background of this gene. In this study, we have reported coexistence of *bla*_KPC-2_ and *bla*_NDM-1_with different genetic context in *P*. *aeruginosa* in a tertiary referral hospital of India.

## Materials and Methods

### Clinical isolates

A total number of 88 non duplicate carbapenem nonsusceptible *P*. *aeruginosa* were collected from the patients admitted to different wards or attended clinics of Silchar Medical College and Hospital, Silchar, India for a period of one year from July 2012 to June 2013 (S1 Table). All the isolates were subjected to PCR assay for detection of class A and class B carbapenemases as described below.

### Molecular characterization & cloning

Multiplex PCR assay was performed for characterizing the *bla* genes using the primers as mentioned (Table 1) and the reaction conditions as described previously [5–7]. The amplicons were sequenced to confirm the variant of amplified gene. For cloning of *bla*_KPC-2_, primers were designed (Table 1) from the flanking region of *bla*_KPC-2_ to amplify the whole gene including the promoter region. The amplified products were purified using MinElute PCR Purification Kit (Qiagen, Hilden, Germany) and was ligated into pGEM-T Vector (Promega, Madison, USA), transformed into *E*. *coli* JM107. Coexistence of class B β-lactamase was determined by multiplex PCR targeting IMP, VIM and NDM gene (Table 1) as described earlier [8–9].

**Table 1:** Oligonucleotides used as a primers in the study.

Target	Primer Pairs	Sequences	Product size	Reference
KPC	KPC-F	5^’^-CATTCAAGGGCTTTCTTGCTGC-3^’^	538	5
	KPC-R	5^’^-ACGACGGCATAGTCATTTGC-3^’^		
Whole KPC gene	KPC-2FW	5^’^-AACCCGATGTGTGCCCATCCG-3’	1070	This study
	KPC-2RV	5’-GCGGCGGTGGTGGGCCAATAG-3’		
IMI/NMC	IMI/NMC-F	5’-CCATTCACCCATCACAAC-3’	440	6
	IMI/NMC-R	5’-CTACCGCATAATCATTTGC-3’		
SME	SME-F	5’-AACGGCTTCATTTTTGTTTAG-3’	831	7
	SME-R	5’-GCTTCCGCAATAGTTTTATCA-3’		
VIM	VIM-F	5’-GATGGTGTTTGGTCGCATA-3’	390	8
	VIM-R	5’-CGAATGCGCAGCACCAG-3’		
IMP	IMP-F	5’-TTGACACTCCATTTACDG-3’	139	8
	IMP-R	5’-GATYGAGAATTAAGCCACYCT-3’		
NDM	NDM-F	5^’^-GGGCAGTCGCTTCCAACGGT-3^’^	476	9
	NDM-R	5^’^-GTAGTGCTCAGTGTCGGCAT-3^’^		
Int1	Int1-F	5^’^-CAGTGGACATAAGCCTGTTC-3^’^	166	10
	Int1-R	5^’^-CCCGAGGCATAGACTGTA-3^’^		
Int2	Int2-F	5^’^-TTGCGAGTATCCATAACCTG-3^’^	288	10
	Int2-R	5^’^-TTACCTGCACTGGATTAAGC-3^’^		
Gene cassette	5CS	5^’^-GGCATCCAAGCAGCAAG-3^’^	-	10
	3CS	5^’-^AAGCAGACTTGACCTGA-3^’^		

### Determination of genetic environment

Integron carriage was assessed by performing integrase gene PCR [10] targeting integrase gene of class 1 and class 2 integron. To determine the cassette array within class 1 integron, four sets of PCR reactions were performed, in first set, primers of 5^/^ conserved region (5CS) and reverse primer of *bla*_KPC_ (KPC-R) were used. In second PCR, a set of primers targeting 5^/^ conserved region (5CS) and forward primer of *bla*_KPC_ (KPC-F) were used. In the third reaction, 3^/^ conserved region (3CS) of class 1 integron and forward primer of *bla*_KPC_ (KPC-F) were taken and the fourth PCR assay was performed using 3CS primer and reverse primer of *bla*_KPC_ (KPC-R). All the primers are mentioned in the Table 1. The PCR conditions were as described previously [5, 10]. The visible amplification was observed in 2^nd^ and 4^th^ PCR assay for both the samples. The amplified products were sequenced and were analyzed using the software available on National Centre of Biotechnology Information website (http://www.ncbi.nlm.nih.gov) and the sequencing results could confirm the location and cassette array of *bla*_KPC_ within class 1 integron. The whole class 1 integron gene cassette was also amplified using primers 5CS and 3CS with the following reaction condition, initial denaturation at 95°C for 3mins, 34 cycles at 95°C for 30s, 49°C for 1min, 72° for 3mins and final extension at 72°C for 7 mins [11].

### Determination of *att*C site

To confirm the presence of *bla*_KPC-2_ within integron, 59 base element PCR assay was performed using the amplified integron harboring *bla*_KPC-2_ as template using HS286 (5^/^-GGGATCCTCSGCTKGARCGAMTTGTTAGVC-3^/^) and HS287 (5^/^-GGGATCCGCSGCTKANCTCVRRCGTTAGSC-3^/^) as described earlier [12]. Each amplified cassette was cloned into pGEM-T Vector (Promega, Madison, USA) and sequenced.

### Plasmid analysis and Southern blot hybridization

Plasmids were purified by Gene Jet plasmid Miniprep kit (Thermo Scientific, Lithuania) and were subjected to transformation by heat shock method using *E*.*coli* JM107 as recipient. Transformants were selected on LB agar with 100μg/ml of ampicillin and 0.25μg/ml of imipenem. Southern blotting was performed on agarose gel by in-gel hybridization with the *bla*_KPC_ and *bla*_NDM_ probe labeled with digoxigenin DIG-high prime labeling mix (Roche, Germany) detection Kit. Plasmid DNA from transformants was separated by pulsed field gel electrophoresis (PFGE) (CHEF DR-III System, Bio-Rad; USA) and was transferred to nylon membrane (Hybond N, Amersham, UK), hybridized with prepared probes. Detection was performed using NBT color detection Kit (Roche, Germany).

### Restriction digestion of plasmid encoding *bla*_KPC-2_

Plasmids of the transformants were digested with *Xba*I, *Eco*R1 and *Hind*III separately. The digested products were separated by PFGE for 14 hour at 4 V/cm at an included angle of 120° and band patterns were analyzed. Further, the fragments were cloned in pGEM-T Vector (Promega, Madison, USA) as per manufacturer’s instruction and were sequenced.

### Plasmid incompatibility typing

Plasmids harboring the resistance determinants were characterized by PCR based replicon typing to identify the different incompatibility (Inc) groups viz. FIA, FIB, FIC, HI1, HI2, I1/Iγ, L/M, N, P, W, T, A/C, K, B/O, X, Y, F and FIIA as described previously [13].

### Antibiotic susceptibility

The antibiotic susceptibility was done by Kirby Bauer disc-diffusion method against antibiotics viz. piperacillin-tazobactam (100/10 μg), amikacin (30 μg), gentamicin (10 μg), ciprofloxacin (5 μg), polymixin B (300 units), netilmicin (30 μg) and carbenicillin (100 μg) (Hi-Media, Mumbai, India). Minimum inhibitory concentration was performed by agar dilution method against imipenem, ertapenem, meropenem, cefepime & aztreonam and the results were compared with standard CLSI guidelines [14]. The antibiotic susceptibility of the transformants and clone of *bla*_KPC-2_ was also determined.

### Strain typing

The heterogeneity in the isolates was determined by Pulse field gel electrophoresis (PFGE) as well as by repetitive extragenic palindromic (REP) PCR and enterobacterial repetitive intergenic consensus (ERIC) PCR [15]. Pulse field gel electrophoresis was performed using *Xba*1 restriction enzyme and the DNA fragments were separated with a CHEF-DR III apparatus for 22 hours at 4 V/cm.

## Results

**Fig 1:**
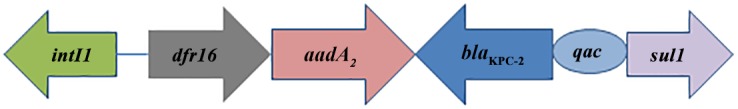
Genetic context of *bla*_KPC_.

Out of 88 *P*. *aeruginosa* tested, two were harboring *bla*_KPC_ gene, confirmed as encoding KPC-2. The first isolate (PA-529) was obtained from a 13 years-old male patient, who attended the clinic in September 2012 and was diagnosed with urinary tract infection and the second isolate (PA-551) was collected from pus sample of 18-years-old female patient admitted to surgery ward in November 2012. Restriction analysis and sequencing revealed KPC-2 was present in two different locations within the host. In first, it was not associated with any mobile genetic element, whereas the second one was class 1 integron mediated attaining reverse orientation (Fig 1). Furthermore, 59 base element PCR confirmed *bla*_KPC-2_ was flanked by *att*C site. *bla*_KPC-2_ was also flanked by *dfr*16 and *aadA2* in the upstream region while *qac* was located in the downstream area within the cassette. In both the isolates, *bla*_KPC-2_ was horizontally transferable as when transformants were selected with ampicillin and subsequently confirmed by Southern hybridization assay. An approximate 35 kb plasmid of Inc F type was isolated and restriction map of plasmids isolated from both the strains showed similar band pattern. Additionally, both the isolates were co-harboring *bla*_NDM-1_. This gene could not be selected with ampicillin but selection with imipenem could successfully transfer NDM-1 and the gene was found to be located in Inc N type plasmid of approximately 26 kb in size. Interestingly, selection with imipenem could only select *bla*_NDM-1_ and not *bla*_KPC-2_. The 35 kb plasmid was hybridized only with *bla*_KPC-2_ probe. MIC results (Table 2) were indicative that *bla*_KPC-2_ was responsible for ertapenem resistance. Besides carbapenem resistance, PA-529 also showed resistance to co-trimoxazole, amikacin, gentamicin, netilmicin, ciprofloxacin and polymixin B whereas PA-551 was found to be susceptible towards gentamicin and polymixin B. PFGE pattern showed both the isolates were of different pulse types and which was also supported by ERIC and REP PCR results where two different clones were observed. In remaining test samples, carriage of *bla*_NDM-1_ was also observed in nine isolates and 16 were phenotypically positive for carbapenemase by modified Hodge test where molecular basis of carbapenem resistance could not be established by our target primers.

**Table 2:** MIC (μg/ml) of the KPC-2 harboring isolates, their transformants and clones.

Organisms	Imipenem	Ertapenem	Meropenem	Cefepime	Aztreonam	Ampicillin
PA-529 (*bla*_KPC-2_ & *bla*_NDM-1_)	>256	>256	>256	>256	>256	>512
*E*. *coli* JM107 (p^KPC-2^/529)	4	16	1	32	32	128
*E*. *coli* JM107 (p^NDM-1^/529)	32	64	16	64	64	128
*E*. *coli* JM107 (Clone *bla*_KPC-2_/529)	2	8	.25	8	4	64
PA-551 (*bla*_KPC-2_ & *bla*_NDM-1_)	>256	>256	128	>256	>256	>512
*E*. *coli* JM107 (p^KPC-2^/551)	2	8	1	16	32	128
*E*. *coli* JM107 (p^NDM-1^/551)	32	32	16	64	32	256
*E*. *coli* JM107 (Clone *bla*_KPC-2_/551)	1	8	.25	4	2	64
*E*. *coli* JM107	< .125	< .125	< .125	< .125	< .125	.125

## Discussion

KPC producing organisms have been described quite often from different parts of the world and more frequently from South America, US and Europe [16]. Carriage of KPC within an unnatural host i.e. *P*. *aeruginosa* is less frequently reported [2] as from India it has been on the record only from *E*. *coli*, *K*. *pneumoniae* and *Proteus mirabilis* [1]. Integron borne KPC is not very common around the world and there are few studies on the record that claim their presence within the gene capture mechanism [17]. Thus, our study suggests integron as a tool for horizontal dissemination of this resistance determinant in our hospital setting. However, the role of *bla*_KPC-2_ exhibiting beta-lactamase activity in reverse orientation is not clear and possibly the other copy of the same gene as observed in the study could play a role in carbapenem resistance. We have also recorded coexistence of *bla*_NDM-1_ in both the isolates as well as resistance towards most of the tested antibiotics, particularly indicates the selection pressure of different groups of beta-lactam antibiotics in the hospital environment of this part of the world. The antibiogram pattern was supported by the existing flanking resistance determinants of *bla*_KPC-2_ within integron conferring a phenotype with trimethoprim, quinolone and aminoglycosides resistance. In this study, it was observed that both the resistance determinants (*bla*_KPC-2_ and *bla*_NDM-1_) were located in two different genetic vehicles. Previous studies have already pointed out the need of investigation on extent of spread of KPC and its role in carbapenem resistance in India [18]. However, recent reports have been from western and southern part of India [3, 18], we identified their expansion in northeastern part of this country as well. This study also established that co-acquisition or subsequent acquisition of *bla*_KPC-2_ and *bla*_NDM-1_ contributed a complete carbapenem resistance in this hospital setting.

## Conclusion

To the best of our knowledge this is the first report of integron borne KPC-2 producing *P*. *aeruginosa* in India. This study advocates implementation of proper antimicrobial policy and infection control management in hospital settings so as to prevent horizontal spread of this resistant determinant within different host range.

## Ethical Approval

The work was approved by Institutional Ethical committee of Assam University, Silchar vide Reference Number: IEC/AUS/C/2014-001. The authors confirm that participants provided their written informed consent to participate in this study.

## Supporting Information

S1 Table Clinical history of patients & details of the samples collected for this study. No ethical, third party or legal restriction limit exists in sharing the data.
(DOCX)
