# Supplementary material for: Co-Carriage of bla KPC-2 and bla NDM-1 in Clinical Isolates of Pseudomonas aeruginosa Associated with Hospital Infections from India
Source: PLoS One. 2015 Dec 29;10(12):e0145823. doi: 10.1371/journal.pone.0145823 (PMC4694648; doi:10.1371/journal.pone.0145823)
Supplement: S1 Table — No ethical, third party or legal restriction limit exists in sharing the data. (DOCX) [file pone.0145823.s001.docx]

**S1 Table: Clinical history of patients & details of the samples collected for this study**

| **Serial number** | **Strain ID** | **Patient’s**  **Sex** | **Patient’s**  **Age** | **Sample origin** | **Ward** |
| --- | --- | --- | --- | --- | --- |
| 1 | PA-19 | Female | 12.5 years | Stool | Medicine |
| 2 | PA-27 | Male | 32 years | Stool | Medicine |
| 3 | PA-28 | Male | 27 years | Surgical wound | Surgery |
| 4 | PA-31 | Male | 62 years | Stool | OPD |
| 5 | PA-32 | Female | 48 years | Urine | OPD |
| 6 | PA-39 | Female | 10 years | Urine | Genital ward |
| 7 | PA-42 | Male | 18.5 years | Throat swab | ENT |
| 8 | PA-47 | Male | 48 years | Pus | OPD |
| 9 | PA-63 | Female | 8.5 years | Ear swab | ENT |
| 10 | PA-76 | Male | 47 years | Pus | Surgery |
| 11 | PA-81 | Male | 20 years | Endotracheal aspirates | ICU |
| 12 | PA-97 | Male | 26.5 years | Cerebrospinal fluid | ICU |
| 13 | PA-119 | Female | 38 years | Blood | Surgery |
| 14 | PA-120 | Male | 11 years | Urine | Genital ward |
| 15 | PA-123 | Male | 42 years | Pus | Surgery |
| 16 | PA-141 | Female | 21 years | Urine | Medicine |
| 17 | PA-143 | Male | 10 years | Throat swab | ENT |
| 18 | PA-179 | Male | 9 years | Blood | Surgery |
| 19 | PA-184 | Male | 23 years | Nasopharyngeal swab | ENT |
| 20 | PA-187 | Female | 7 month 10 days | Stool | OPD |
| 21 | PA-188 | Male | 37 years | Surgical wound | Surgery |
| 22 | PA-191 | Male | 41 years | Pus | Surgery |
| 23 | PA-201 | Male | 23 years | Urine | Medicine |
| 24 | PA-203 | Male | 29 years | Urine | Medicine |
| 25 | PA-214 | Male | 76 years | Stool | OPD |
| 26 | PA-236 | Male | 22 years | Sputum | OPD |
| 27 | PA-237 | Male | 61.5 years | Stool | OPD |
| 28 | PA-249 | Female | 83 years | Endotracheal aspirates | ICU |
| 29 | PA-253 | Male | 70 years | Surgical wound | Surgery |
| 30 | PA-267 | Female | 45 years | Pus | Surgery |
| 31 | PA-271 | Female | 37 years | Stool | Medicine |
| 32 | PA-278 | Female | 25 years | Urine | Medicine |
| 33 | PA-279 | Male | 35 years | Wound aspirate | Surgery |
| 34 | PA-282 | Female | 19 years | Urine | Medicine |
| 35 | PA-287 | Male | 29 years | Sputum | Medicine |
| 36 | PA-288 | Male | 11 years | Stool | OPD |
| 37 | PA-299 | Female | 59 years | Urine | Medicine |
| 38 | PA-301 | Female | 27 years | Sputum | OPD |
| 39 | PA-303 | Female | 14.5 years | Urine | Medicine |
| 40 | PA-308 | Female | 42 years | Pus | Surgery |
| 41 | PA-311 | Male | 56 years | Surgical wound | Surgery |
| 42 | PA-318 | Female | 19 years | Urine | Medicine |
| 43 | PA-329 | Male | 79 years | Stool | Medicine |
| 44 | PA-332 | Female | 66 years | Stool | Medicine |
| 45 | PA-347 | Female | 31years | Urine | Medicine |
| 46 | PA-358 | Male | 34 years | Stool | OPD |
| 47 | PA-365 | Male | 29 years | Ear swab | ENT |
| 48 | PA-379 | Male | 36 years | Pus | Surgery |
| 49 | PA-381 | Female | 67 years | Blood | Surgery |
| 50 | PA-387 | Male | 55 years | Oral swab | Medicine |
| 51 | PA-402 | Male | 51 years | Pus | Surgery |
| 52 | PA-407 | Male | 44 years | Urine | Medicine |
| 53 | PA-415 | Female | 59 years | Stool | Medicine |
| 54 | PA-436 | Male | 34 years | Stool | OPD |
| 55 | PA-437 | Male | 56.5 years | Sputum | Medicine |
| 56 | PA-458 | Male | 48 years | Pus | Medicine |
| 57 | PA-464 | Male | 9 years | Oral swab | OPD |
| 58 | PA-496 | Male | 12 years | Stool | Medicine |
| 59 | PA-497 | Female | 1 year 2 months | Stool | OPD |
| 60 | PA-518 | Male | 16 years | Pus | Surgery |
| 61 | PA-529 | Male | 13 years | Urine | OPD |
| 62 | PA-531 | Male | 38 years | Cerebrospinal fluid | ICU |
| 63 | PA-535 | Male | 66 years | Stool | Medicine |
| 64 | PA-539 | Female | 71 years | Pus | OPD |
| 65 | PA-542 | Male | 23 years | Stool | Medicine |
| 66 | PA-544 | Female | 8 years | Blood | Surgery |
| 67 | PA-547 | Female | 15 years | Sputum | Medicine |
| 68 | PA-550 | Female | 22 years | Urine | Medicine |
| 69 | PA-551 | Female | 18 years | Pus | Surgery |
| 70 | PA-558 | Female | 33 years | Wound aspirate | Surgery |
| 71 | PA-562 | Female | 53.5 years | Pus | Surgery |
| 72 | PA-569 | Female | 67 years | Stool | Medicine |
| 73 | PA-583 | Female | 52 years | Pus | Surgery |
| 74 | PA-584 | Male | 68 years | Wound aspirate | Surgery |
| 75 | PA-588 | Male | 33 years | Ear swab | ENT |
| 76 | PA-601 | Male | 17 years | Urine | OPD |
| 77 | PA- 612 | Male | 20 years | Pus | Surgery |
| 78 | PA-617 | Female | 31 years | Pus | Surgery |
| 79 | PA-623 | Female | 29 years | Urine | Medicine |
| 80 | PA-624 | Male | 47 years | Urine | Medicine |
| 81 | PA-638 | Female | 43 years | Throat swab | ENT |
| 82 | PA-639 | Male | 19 years | Pus | Surgery |
| 83 | PA-642 | Female | 26 years | Urine | OPD |
| 84 | PA-648 | Male | 57 years | Surgical wound | Surgery |
| 85 | PA-652 | Female | 21 years | Urine | Medicine |
| 86 | PA-675 | Male | 14 years | Blood | Surgery |
| 87 | PA-679 | Male | 63 years | Pus | Surgery |
| 88 | PA-683 | Male | 56 years | Stool | Medicine |
